# Feedback Enhances Feedforward Figure-Ground Segmentation by Changing Firing Mode

**DOI:** 10.1371/journal.pone.0021641

**Published:** 2011-06-28

**Authors:** Hans Supèr, August Romeo

**Affiliations:** 1 Institute for Brain, Cognition and Behavior (IR3C), Barcelona, Spain; 2 Dept Basic Psychology, Faculty of Psychology, University of Barcelona, Barcelona, Spain; 3 Catalan Institution for Research and Advanced Studies (ICREA), Barcelona, Spain; Indiana University, United States of America

## Abstract

In the visual cortex, feedback projections are conjectured to be crucial in figure-ground segregation. However, the precise function of feedback herein is unclear. Here we tested a hypothetical model of reentrant feedback. We used a previous developed 2-layered feedforwardspiking network that is able to segregate figure from ground and included feedback connections. Our computer model data show that without feedback, neurons respond with regular low-frequency (∼9 Hz) bursting to a figure-ground stimulus. After including feedback the firing pattern changed into a regular (tonic) spiking pattern. In this state, we found an extra enhancement of figure responses and a further suppression of background responses resulting in a stronger figure-ground signal. Such push-pull effect was confirmed by comparing the figure-ground responses withthe responses to a homogenous texture. We propose that feedback controlsfigure-ground segregation by influencing the neural firing patterns of feedforward projecting neurons.

## Introduction

Figure-ground segmentation refers to the assignment of visual elements to either objects or background and is a primary step in visual perception. In the brain, visual features are detected by neurons by means of their feedforward defined classical receptive field whereas contextual influences beyond the classical receptive field have been interpreted as the neural substrate of figure-ground segmentation. In the primary visual cortex (V1), cortical feedback projections covering large parts transmit extra-classical receptive field information [1] and are considered to be critical for figure-ground segmentation. This assumption is reflected in many theoretical [e.g. 2,3] and computational models that explain figure-ground segregation by recurrent processing through horizontal and/or feedback connections [4]–[19].

However the exact role of feedback in figure-ground segregation is not clear. For instance has feedbacka decisive role in the occurrence of figure-ground activity or more modulatory role incontrolling the strength of the figure-ground signal? Consistent with the former role, visual context presumably transmitted by feedback may activate non-stimulated regions of V1 [20], and in agreement with TMS experiments [e.g. 21,22; see also 23], patient studies demonstrate that V1 alone is not sufficient for simple figure-ground segregation[24]suggesting that feedback is required. Yet other arguments are inconsistent with a leading role of feedback projections in producing contextual effects and figure-ground segmentation. Inactivation of V2, which is the main contributor of feedback to the primary visual cortex, has no effect on centre-surround interactions of V1 neurons [25].A lesion study provides further evidence showing that after removing most of the feedback (including V3, V4, MT, MST, but not V2) to V1detection of textured figure-ground stimuli presented in the lesioned field was not affected [26]. This means that figure-ground segmentation occursin parts of the cortex that donot receive feedback.

Recently using computer modeling, we have demonstrated that figure–ground segregation can be achieved in a purely feedforward way [27]–[28]. In other studies we showed that thestrength of figure-ground modulation to a particular stimulus is not fixed but depends on the state of V1 neurons [29]–[31]. Cortical state that is characterized by the way neurons fire, i.e. burst versus tonic firing, controls the transmission of feedforward information [32], [33]. Thisdifferential gating of feedforward information involves inhibition by feedback projections [32]–[35].Taking these findings together, we therefore speculate that a possible role for feedback is to control the strength of the figure-ground signal by influencing the cortical state.

To test this ideawe used our previous described computer model [27]. Our data show that without feedback, neurons respond with low-frequency (∼9 Hz) bursting to a figure-ground stimulus. Feedback changed this firing pattern into a tonic spiking pattern. In this state,a further enhancement of the responses to the figure and a further suppression of background responses were observed resulting in a stronger figure-ground signal.To be effective, surround inhibition must arrive after but within 100 ms, the feedforward induced responses. Such push-pull effect, which appears to be typical in figure-ground segregation[36]–[38], was confirmed by comparing the figure-ground responses with the responses to a homogenous texture. In conclusion, we propose that feedback controls the segregation of figure from ground by influencing the neural firing patterns of feedforward projecting neurons.

## Results

**Figure 1:**
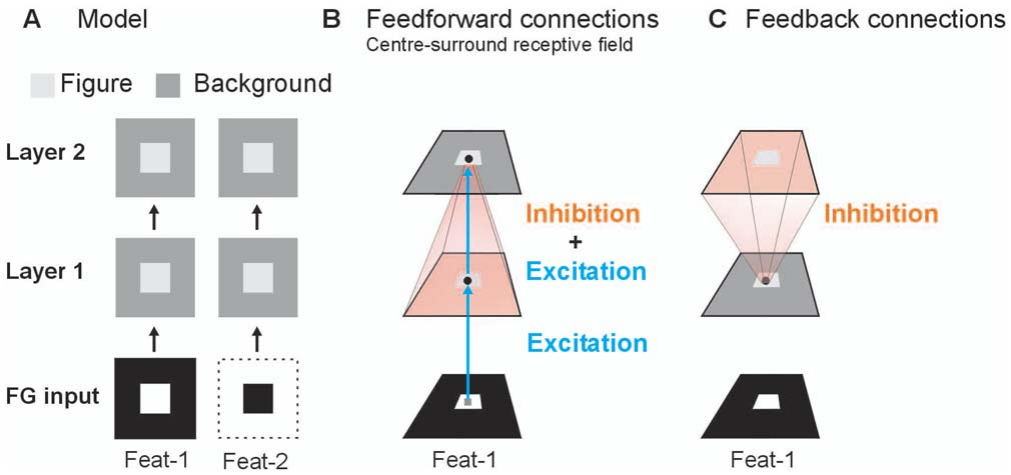
Model, receptive field organization and figure-ground segregation. **A**: The model consists of two separate feature channels (Feat-1 and Feat-2) with each two layers, which are unidirectional connected (arrows). The white regions in the two lower squares indicate the stimulus input (figure-ground input). Black regions provide no input to the model. In the two layers of the model, the light grey central squares depict the figureregion and dark grey regions the background. **B**: Layer-1 neurons have a centre receptive field, i.e. they are driven by one input pixel. Layer-2 neurons have an excitatory centre and inhibitory surround receptive field. The central small black circles represent a neuron in the first and second layer of the model. The small grey square represents one input pixel. Blue arrows indicate point-to-point (retinotopic), excitatory connections and orange region represent the inhibitory connections from layer 1 to layer 2.**C**: Each layer-1 neurons receives inhibitory feedback connections from all layer-2 neurons, indicated by the orange region. Connections are identical for both feature maps. Note that in B,C only one feature map is shown.

**Figure 2:**
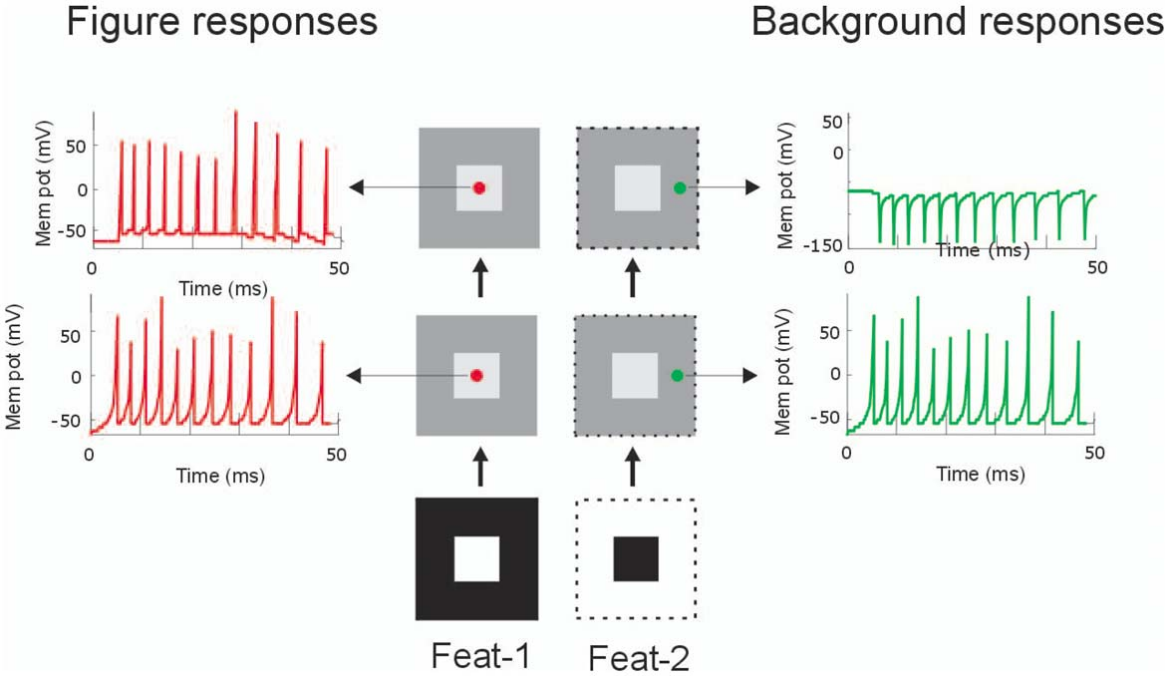
Spike responses of the neurons in the first and second layer to figure-ground stimulus. Arrows point to the responses of neurons (small circles) lying on the figure (red traces) and background (green traces) regions.

We employed our earlier designed 2-layered model of spiking neurons [27]using an input design (fig. 1a) for modeling figure-ground segregation [9], [16]. The model consist of two feature channels (Feat-1 & Feat-2), which represent two neuronal cell populations with opposite preference for a single feature. The model input not only corresponds to texture defined images but also to luminance, direction of motion, color defined figures. Neurons in layer 1 transformed by means of their point-to-point excitatory connections (fig. 1b) the figure-ground input into a spike map. These neurons responded with a transient burst of 12 spikes (fig. 2). The layer-2 neurons integrated this information through their centre-surround receptive fields (fig. 1b). As a result in the first feature channel (Feat-1), neurons at the central figure location produced a similar spike burst as layer-1 neurons (fig. 2). In contrast to the Feat-1 condition, neurons in the second feature channel (Feat-2) became quiescent (fig. 2). Here the relatively large activated surrounding (background) region provoked a strong suppression neutralizing the point-to-point excitation. Strong inhibition however leads to rebound spiking [27], [28]. As a consequence, in the second layer basic figure-ground segregation by global inhibition was achieved [27,28; see also 39]; neurons located in the central figural region fired spikes while surrounding (background) neurons were silent.This agrees with early studies reporting that V1 neurons generally do not respond to areas of uniform luminance.

**Figure 3:**
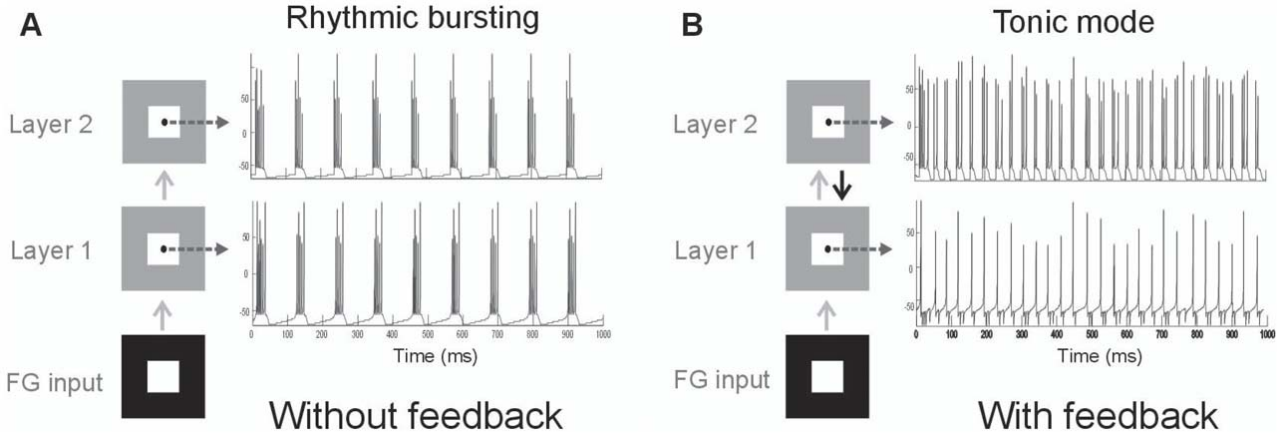
Figure-ground responses. **A, B**: Responses without (A) and with (B) feedback. Small circle represents a single neuron. Time is from stimulus onset.

**Figure 4:**
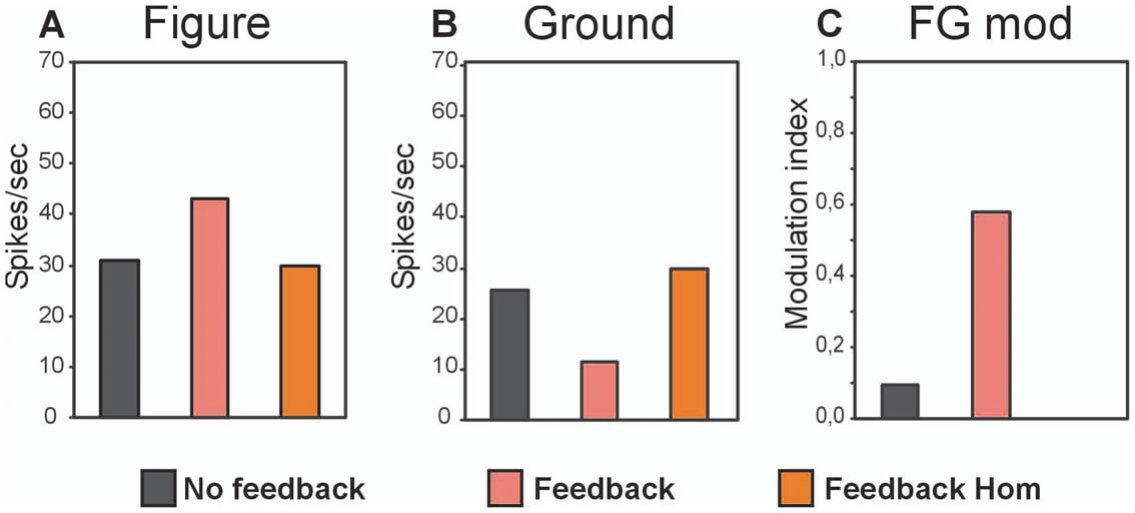
Average responses to figure, ground and homogeneous input (A,B) and strength of figure-ground segmentation (C) with (red/orange) and without (black) feedback connections.

Analyzing the responses over a longer time period (1 sec) showed that a continuous figure-ground input resulted in continuous low-frequency (∼9 Hz) bursting in both layers and conditions (fig. 3a). The response rate (46 spikes/s) of layer 1 neurons was similar for neurons located at the figure and background location (table 1).Thus over longer periods background neurons do respond to the input, which agrees with reports showing that some V1 neurons do respond to uniform surfaces covering their RF [e.g. 40].In the second layer figure-ground segregation occurred where neurons at the figure location responded slightly higher than the neurons at the background location (fig. 4).

**Table 1:** Number of spikes per second of layer-1 neurons in the two feature maps in the presence or absence of feedback.

	Figure	Background
Feat map	No Feedback	Feedback	No Feedback	Feedback
1	46	23	0	0
2	0	0	46	50

### Effect of feedback on figure-ground responses

We then included feedback connections from layer-2 neurons to layer-1 neurons.Feedback changed thelow-frequency bursting firing pattern into a tonic spiking pattern (fig.3b).Feedback had no major effect on the background responses (50spikes/s)of the neurons in layer 1. Figure responses, however, showed a decrease in response rate of 40% (table 1). Despite the lower figure responses of layer 1 neurons, we found that after including feedback the average figureresponses were enhanced and background responses suppressedof the neurons in layer 2 (fig. 4; red bars). Comparing these responses to the responses with the responses to a homogenous texture,an increasedspike ratewas observed for the neurons located at the figure region and a decreased spike rate for the neurons located on the background (fig. 4a,b; orange bars). So, inhibitory feedback produces a stronger figure-ground modulation (fig.4c).

### Changing strength of feedforward and feedback connections

**Figure 5:**
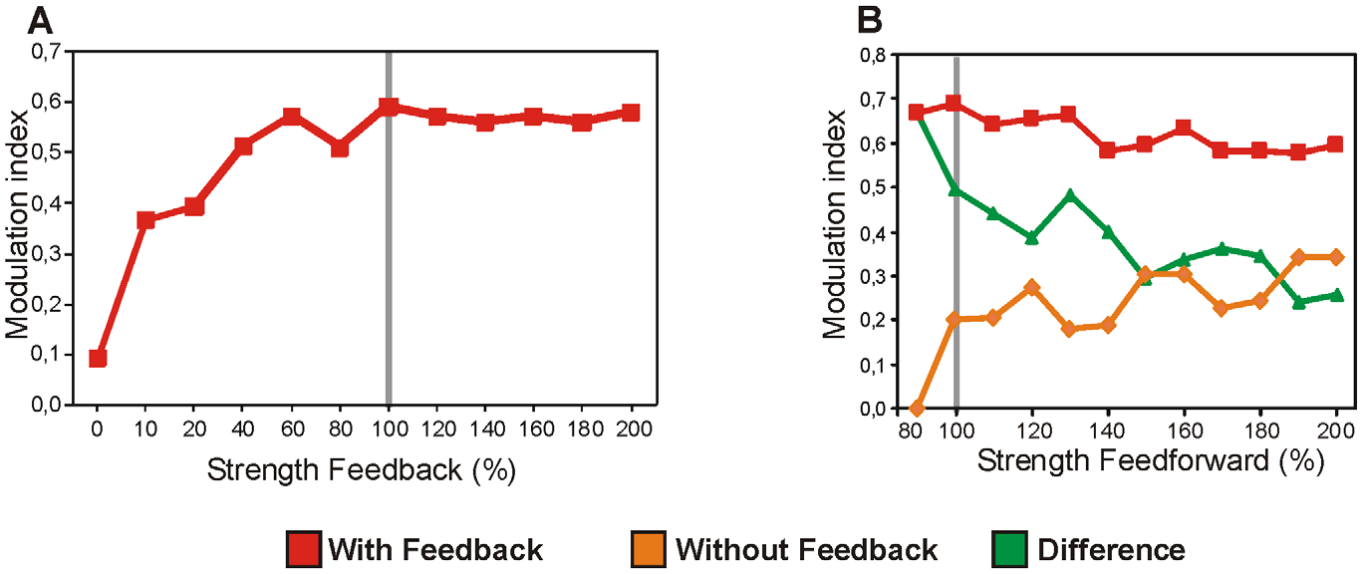
Amount of figure-ground modulation after testing the model with different strengths of feedback connections (A) and feedforward connections (B). Grey lines indicate the strength of figure-ground modulation observed in the previous experiments and is used as reference.

We then changed the weights of the feedback and feedforward connections. Modifying the strength of the feedback connections caused a change in the strength of figure-ground modulation (fig. 5a). Stronger feedback connections (i.e. more inhibition) resulted in anenhancement of the figure-ground signal in layer 2 while weaker feedback connections lead to a decrease in the figure-ground signal. When changing the feedforwardconnections, we observed that figure-ground modulation specifically was enhanced when the feedforward connections were weak (fig. 5b). When feedback was absent the weak (80% of the initial value) feedforward connections did not produce figure-ground activity. When feedback was included strong modulation was observed for the same weak stimulus input (fig. 5b, orange point vs. red point at 80%). Thus feedback specifically enhances figure-ground modulation at lowstimulus contrast, as indicated by the green trace in figure 5.

### Time delay of feedback inhibition

**Figure 6:**
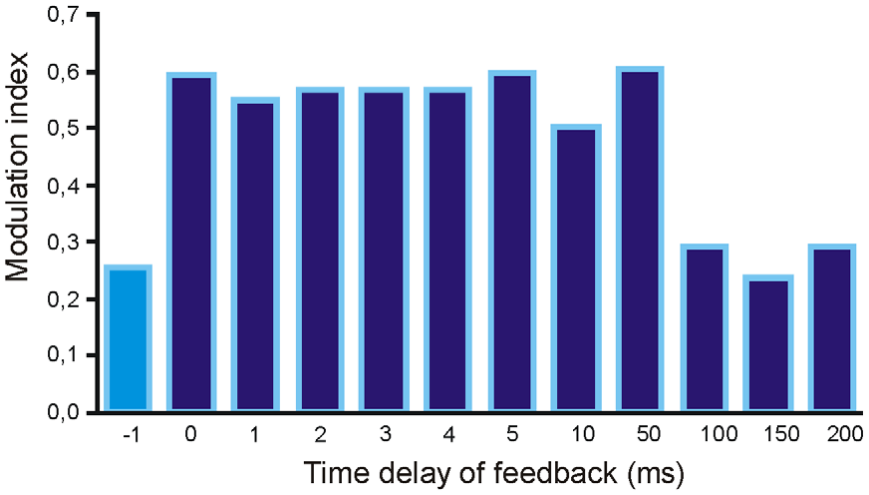
Amount of figure-ground modulation for different time delays of feedback inhibition. Sign indicates whether feedback input arrives before (negative value) or after (positive values) the feedforward input. For each time delay one iteration step should be added.

To better understand why inhibition changes figure-ground segregation, we varied the time of arrival of the spikes from layer 2 to layer 1. The resultsshow that feedback inhibition producedstronger FG segregation when it arrived after, and not before, the feedforward evoked spikes (fig. 6). Feedback input however must arrive within 100 ms to be effective.When feedback inhibition came later than 100 ms, FG modulation was not enhanced. Thus inhibition influences the dynamic behavior of the spiking neuron within a limited but relative long time interval.

## Discussion

In this study, we showedby means of a simple 2-layered spiking network that feedback increases the feedforward segmentation of figure from background elements by enhancing the figure responses and at the same time lowering the background responses.To do so, inhibitory feedback changed the responsefrom bursting to tonic mode and did not activate neurons preventing the model from going into an open-loop.

### Feedback

Feedback connections from extra-striate areasshow an orderly topographic organization and terminate in discrete patches within V1. These patchy feedback terminations overlap with patches of V1 feedforward projecting neurons [1]. Furthermore, feedback tends to target alike tuned cells [41], and correlate with ocular dominance, iso-orientation columns, and CO blobs [42].Together with the described wide spread termination in V1, feature selectivity of feedback was incorporated in our model architecture.

In the visual system, the contribution of surround is asymmetric with a shape that is related to the feature selectivity of the target cell [e.g. 43]. We have simplified this notion by feature maps and feature selective inhibition. In our model inhibitory feedback onto a layer-1 neuron comes from all layer-2 neurons. This signifies that the surround is a fixed term and is not shape dependent. The only relevant factors of the surround are size and time. Thus, the different types of surrounds observed in early visual areas [e.g. 43,44] are amalgamated into a difference in the balance between excitation and inhibition. Thus although on the first sight our model may be (too) simple, it nevertheless captures the essential elements of centre-surround processing.

In our model feedback arrived almost immediately to the ascending neurons. The almost immediate effect of V2 feedback on their target neurons in V1, where it acts on the first stimulus evoked spikes [45] agrees with this feature. Such a fast effect is indicative for direct feedback onto the ascending V1 neurons. For instance, the same V1 layers that send ascending signals to extra-striate areas, e.g. V2 or MT, receive information back from these areas. However, unlike our model there is no clear evidence (yet) of re-entrant feedback to the visual cortex. On the contrary, inactivation of V2, which is the main contributor of feedback to the primary visual cortex, has no effect on centre-surround interactions of V1 neurons [25]. This finding contrasts the interpretation that inhibitory feedback in our model represents V2 feedback. Alternatively, surround inhibition may derive from the wide spread lateral connections that exists in the visual cortex.Intrinsic inhibitory connections convey information from beyond the classical receptive field and can provide surround information of the target stimulus.It has been shown that contextual suppressive effects come from large regions (4-7 mm). According to our findings inhibitory feedback needs to arrive within 100 ms after the feedforward evoked response. Such relatively long time interval may overcome the rather slow conductance velocities (typically 0.1-0.2 m/sec) observed forlateral fibers.

### Feedback: a push-pull mechanism to enhance stimulus contrast

In our model feedback has a direct consequence on the activity of the ascending neurons where it lowers the responses to figure elements in layer 1. Despite the inhibitory nature, feedback enhances the figure-ground signal in layer 2.Feedback accomplishes this by a differential effect on neural activity; it enhances figure responses and lowers background responses. Such push-pull effect is also observed in neurons of the visual cortex responding to figure-ground textures [36]–[38].Moreover, we show that feedback especially enhances figure-ground signal when the feedforwardinput is relatively weak (see fig. 4b).So feedback acts as a kind of attention mechanism enhancing stimulus contrast [46], [47]. In accordance, feedback improves stimulus response precision [48] and feature contrast [49], and enhances figure-ground discrimination [50], and top-down attention may enhance feedforward responses in the LGN [51] and figure-ground modulatory responses in early cortex [52]–[54].Therefore, instead of generating the contextual effectsneeded for figure-ground segmentation, we speculate that inhibitory feedback boosts the feedforward generated figure-ground activity. Markedly, feedforward inhibition decreases the figure-ground signal [27] whereas inhibition through feedback increases the figure-ground signal [current study]. Further studies are needed to understand the dynamics that lead to such a difference.

### Cortical state, attention, and figure-ground segmentation

The strength of figure-ground modulation depends on the momentary state of the visual cortex[29]–[31]. A proper state is characterized by low-frequency correlated neural firing. Absence or deficiency in such synchronous firing prohibits figure-ground segregation resulting in the occasionally failure to detect a stimulus [29]. In this study, we show that feedback affects the strength of figure-ground activity by changing the cortical state, i.e. changing the firing from low-frequency bursting mode (9 Hz) to a tonic firing pattern, which is consistent with the observations that feedback shifts neural responses in the thalamus from a bursting mode into a tonic mode [32].Low frequency or busting activity is generally associated with less attentive states. For example, in the thalamic LGN of the awake animal, bursting is more common during periods of drowsiness and is largely restricted to episodes lasting a few seconds with most of the episodes showing rhythmic bursting activity in the delta (0.5–4 Hz) frequency[55]. In accordance, other studies report that the state of vigilance is associated with single or tonic firing patterns whereas rhythmic bursting at alpha frequencies (8–12 Hz) relates to periods of low vigilance[56], [57].Furthermore, tonic firing increases the signal-to-noise ratio [32]. Similarly to the dynamical changes in cortical state, fast temporal changes in EEG activity have also been associated with changes in attention and discrimination [58]–[60].Putting these findings together it is reasonable to assume thatmoments of high vs. low vigilance,so to say, have different strength of figure-ground modulationbecause of the different firing pattern of the ascending neurons[see also 29].

Such an explanation may also be relevant for the observed discrepancy on attentional effects in V1. Whereas single-unit studies of attention in monkeys have repeatedly revealed relatively modest attentional modulations in V1, human functional magnetic resonance imaging studies demonstrate a large attentional enhancement of the blood oxygen level-dependent (BOLD) signal in V1.A recent report shows that the neuronal metabolic rate differs between low frequency oscillatory bursting and more random or aperiodic (tonic) neural firing where the former gives smaller BOLD responses[61].If one considers that attention, carried by top-down feedback, affects besides spike rate also the firing pattern (bursting versustonic) fMRI recordings will measure a stronger attentional signals than single cell recordings.Finally,it has been shown that cognitive processing of sensory stimuli, like attention is represented by spike rate as well as by spike timing (synchrony). The finding that feedback changes spike rate by changing spike timing may shed some new light on the debate about the neural correlates of cognitive processing.

## Materials and Methods

### Model architecture

The model is composed of two feature channels with each two layers (fig. 1a) of NxNneurons of the Izhikevich type [62]. We used N = 64 but lower and higher values of N are also used and did not critically affect model performance. The two separate feature channels represent two neuronal cell populations with opposite preference for a single feature. The channels are referred to as Feat-1 and Feat-2 condition.

### Feedforward and feedback projections

The excitatory feedforward projections from the stimulus input to the first neural layer and from the first to the second neural layer were retinotopic (point-to-point connections) where pixel/neuron N*_ij_*in one layer solely connected to neuron N*_ij_* in the next layer. Thus the excitatory part of a neuron's receptive field had size one. Neurons in the first neural layer did not receive inhibitory signals from the stimulus input. Each neuron in the second layer received inhibition from all neurons located in the preceding layer. Inhibition was achieved by assigning negative weights to the connections. In the feedback condition, each neuron in the first layer received global inhibition from all layer-2 neurons of the same feature channel. Feature selectivity of feedback was chosen because feedback targets alike tuned cells in the visual cortex [41] and correlate with iso-orientation columns [42].

### Stimulus inputs

The studied textured figures were two arrays of N×N pixels, with N as in the model. Input arrays were binary (0 or 1) corresponding to the preference for a single visual feature such as luminance, orientation, direction of motion, color etc. In other words, 1 stands for optimal tuning whereas 0 is the opposite. In the Feat-1 condition stimulus input was defined as an array of zeros except for the centre region of 16×16 pixels where the pixels had a value of 1 [see also 27]. The other array for the Feat-2 condition was its binary complement, which represented the reverse preference of the visual feature. Together they formed the figure-ground texture [9], [16]. The homogenous texture was a matrix in which all pixels had a value of 1.

### Model dynamics

Cell dynamics is described by the spiking model of Izhikevich [62]

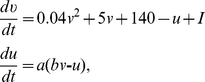
(1) supplemented with the after-spike reset rule 
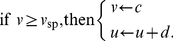
(2)



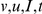
are dimensionless versions of membrane voltage, recovery variable, current intensity and time. Further, *a* is a time scale for *u*, *b* measures the recovery sensitivity, *c* is the reset value for 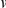, and *d* is the height of the reset jump for 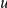. A capacitance factor C was chosen to be 1 and therefore omitted. For all our simulations *a* = 0.02, *b* = 0.25, *c* = −55, *d* = 0.05, and 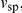 = 30. When dimensions are reintroduced, voltages are read in mV and time in ms.

As initial conditions at *t_0_* = 0 we set
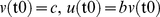
(3)for all the positions in our arrays (since we deal with two-dimensional objects, equations (1) and (2) are actually meant for 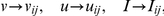 *i,j* = 1, N, and condition (3) is in fact applied to 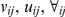. We used the Euler method with 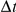 = 0.20 msec. The input current I in (1) is the result of summing different matrix contributions of the form
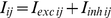
(4)where ‘exc’ stands for ‘excitatory’, ‘inh’ for ‘inhibitory’, and *i,j* are spatial indices.

Further, for neural layers,
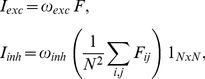
(5)



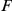
 is either the two dimensional figure itself or the binary array defined by the presence of spikes, i.e., with ones where condition (2) is satisfied and zeros elsewhere. The 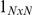 symbol denotes an NxN matrix containing just ones. Since excitatory receptive fields have size one, excitatory signals are point-by-point (retinotopic) copies of 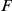
itself, multiplied by the corresponding weight. The inhibitory part, whose associate receptive field has the same size as 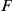, produces a spatially constant term –hence the 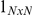 matrix- which is proportional to the normalized sum of all the F coefficients times the inhibitory weight. Thus center and peripheral neurons receive the same amount of inhibition. In our design, the employed weights were 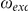 = 1 for the stimulus input to neural layer 1 and 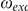 = 400, 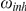 = −700 for the signals from neural layer 1 to neural layer 2. The weight of the feedback connection was 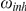 = −50. For strong feedback 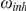 = −100 and for weak feedback 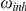 = −10. Different proportions of the feedforward weights, 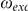, of the stimulus input to neural layer 1 were also tested (see results).

### Calculating responses

To calculate the amount of figure-ground modulation we employed a modulation index (F–G)/ (F+G), where F and G stand for the amount of spikes at the figure and ground regions, respectively [63]. The figure (background) responses from the two central (surround) regions of both feature channels were averaged.
